# Nightmare frequency in last trimester of pregnancy

**DOI:** 10.1186/s12884-016-1147-x

**Published:** 2016-11-09

**Authors:** Michael Schredl, Maria Gilles, Isabell Wolf, Verena Peus, Barbara Scharnholz, Marc Sütterlin, Michael Deuschle

**Affiliations:** 1 Central Institute of Mental Health, Medical Faculty Mannheim/Heidelberg University J5, 68159 Mannheim, Germany; 2 Department of Gynecology and Obstetrics, University Medical Center Mannheim, University of Heidelberg, Theodor-Kutzer-Ufer 1-3, 68167 Mannheim, Germany

**Keywords:** Nightmares, Pregnancy, Stress

## Abstract

**Background:**

Pregnancy-related dreams are often found in pregnant women but also the number of negatively toned dreams seems to be increased in this challenging phase of a woman’s life.

**Methods:**

Nightmare frequency and subjectively experienced stress was elicited via questionnaires. The mothers-to-be were approached during their application visit about 4–8 weeks prior to delivery in three obstetric hospitals. The present analysis included 406 women aged 16–40 years in the last trimester of their pregnancy. Women with severe somatic illnesses and/or psychiatric disorders were excluded. The representative sample included 496 women (age range: 14–93 years.).

**Results:**

The findings clearly indicate that pregnant women report nightmares more often compared to a representative sample and that nightmare frequency is closely related to subjectively experienced stress during daytime. Moreover, baby-related dreams were correlated with nightmare frequency but not with day-time stress.

**Conclusions:**

Future studies should investigate the prevalence of nightmare disorders in pregnancy and study whether brief interventions like Imagery Rehearsal Therapy are beneficial for pregnant women suffering from nightmares.

## Background

Pregnancy, as a period in women’s lives, is characterized by profound changes at all levels: physical, hormonal and psychological [1, 2]. During pregnancy, especially in the third trimester, sleep disorders are quite common [3]: increased snoring (11–16 %), restless legs symptoms (18–31 %), and impaired subjective sleep quality (39–54 %). Not only is sleep altered during pregnancy but dreaming is also affected – with dreaming defined as subjective experiences occurring during sleep [4].

There is a long history of pregnancy-related dreams that can be found in the literature, starting with conceptions dreams, for example, related to the birth of saints [5] and announcing dreams predicting characteristics of the future baby like sex [6]. Perry, DiPietro [7] reported that all 8 women who based their prediction of the baby’s sex on dreams were correct but Maybruck [6] didn’t find above-chance predictions. The majority of studies in this field have shown that the dreams of pregnant women include more often pregnancy-related topics like fetus/baby, being pregnant, childbirth, references to body anatomy [8–17] and, thus, support the continuity hypothesis of dreaming [18]. The continuity hypothesis in its general form states that we dream about the topics that are relevant in our waking-lives [19]. Women during their first pregnancy dreamed more often about their pregnancy than women who have borne more than one child [13]. Interestingly, an increased frequency of pregnancy-related dreams can also be found in expectant fathers [20].

The dreaming process during pregnancy also has, however, its downside: two studies [21, 22] reported a higher frequency of disturbing dreams in pregnant women compared to non-pregnant women. In addition, research indicated that pregnancy-related worries like loss, danger to the fetus/baby or giving birth to a deformed baby also occurred in dreams during pregnancy [23–25]. The number of these negatively toned dreams correlated with day-time depressive mood [26, 27] and trait anxiety [8]; i.e., the dreams including pregnancy-related worries also reflect the day-time worries of pregnant women. Nightmares are defined as extended, extremely dysphoric and well-remembered dreams that usually involve threat to survival, security or physical integrity [28] and, thus, represent an extreme form of negatively toned dreams. About 40–50 % of pregnant women experience nightmares at least sometimes [29] but about 6–10 % of pregnant women reported severe nightmares related to fear of childbirth [30].

The most rigorous study on nightmares during pregnancy was carried out by Lara-Carrasco, Simard [21]. Of the total sample of 57 pregnant women, 32 % reported nightmares once a week or more often (20 % of the non-pregnant control group). Even more pronounced and statistically significant was the percentage of women who reported more than one nightmare per week, (21 % vs. 7 %) - compared to the larger group of women with one nightmare per week and women with more than one nightmare per week. Although, a formal diagnosis of a nightmare disorder [28] was not made, data from representative surveys [31–33] indicate that persons who report one nightmare per week or more often will likely suffer from nightmare disorder. The prevalence rates of the Lara-Carrasco, Simard [21] study, however, have to be viewed with caution as they selected only women with dream recall higher than once per week. As the average dream recall frequency in the population is slightly below one dream per week [34] and dream recall correlates strongly with nightmare frequency [35], it is very likely that the percentages reported by Lara-Carrasco, Simard [21] are biased in the direction of overestimation.

The present study was carried out to compare nightmare frequency in pregnant women compared to a representative sample, controlling for possible differences in dream recall frequency. Furthermore, we were interested in how daytime stress measures were related to nightmare frequency as research in non-pregnant samples clearly indicated a significant relationship between stress and nightmares [35, 36]. Lastly, the hypothesis was tested as to whether pregnant women who report baby-related dreams also report more nightmares based on the findings that worries about the health of the baby also shows up in negatively-toned dreams.

## Methods

### Participants

Overall, 406 women participated in in the POSEIDON study (see procedures). Their mean age was 31.43 ± 5.08 years. (range: 17–44 years.) More than 50 % (*N* = 217) were nulliparous, 189 women were multiparous. The questionnaires were administered on average within the gestation week 36.49 ± 2.38.

A representative sample of 496 women published by Schredl [33] was used for comparison. The mean age of this sample was 49.11 ± 17.97 years. (range: 14–93 years).

### Dream questions

All participants were asked to rate their dream recall during the last months on a 7-point rating scale (0 = never, 1 = less than once a month, 2 = about once a month, 3 = twice or three times a month, 4 = about once a week, 5 = several times a week, 6 = almost every morning). The retest reliability of the scale (mean retest interval: 54.8 ± 44.8 days; *N* = 198) was *r* = .83 [37]. To obtain units of mornings per week, the scale was recoded using the class means (0 → 0, 1 → 0.125, 2 → 0.25, 3 → 0.625, 4 → 1.0, 5 → 3.5, 6 → 6.5). If the person checked “once a week”, the value of the recoded variable was 1 (one morning with dream recall per week). If she checked “several times” a week, the recoded variable was set to 3.5 mornings with dream recall (the mean as the possible range is from 2 to 5).

In addition, nightmare frequency was measured using an 8-point scale (0 = never, 1 = less than once a year, 2 = about once a year, 3 = about two to four times a year, 4 = about once a month, 5 = twice or three times a month, 6 = about once a week, 7 = several times a week) – also showing a high re-test reliability of *r* = .75 [38]. A specific definition of nightmares was not provided. To obtain units in frequency per month, the scale was recoded using the class means (0 → 0, 1 → 0.042, 2 → 0.083, 3 → 0.25, 4 → 1.0, 5 → 2.5, 6 → 4.0, 7 → 18.0), e.g., the nightmare frequency “about once a year” was transformed into 0.083 nightmares per month.

Lastly, one item of the Prenatal Attachment Inventory [39, 40] was included in the present analysis: “I dream about the baby.” The answer categories were: “almost never (0)”, “sometimes (1)”, “often (2)” and “almost always (3)”.

### Stress questionnaires

The German NEO Five-Factor-Inventory comprising 30 items was used to measure trait neuroticism [41]. The internal consistency (Cronbach’s alpha) of the neuroticism score (mean of 6 four-point items) is high (*r* = .81), comparable with the long version (60 items of the questionnaire). The Perceived Stress Scale (PSS) measures subjective stress experiences regarding situations in daily life [42]. The sum score of the 14 items showed high retest reliability and high internal consistency (both r_tt_ > .80). The trait version of the State-Trait-Anxiety Inventory (STAI) encompasses 20 four-point statements regarding emotional and cognitive aspects of anxiety [43]. The total sum score ranging from 20 to 80 showed high internal constancy (*r* = .92). The Edinburgh Postnatal Depression Scale (EPDS) was originally developed to assess postnatal depression but has also been validated for eliciting depressive mood during pregnancy [44, 45]. The sum score of the ten items of the German version [46] showed high reliability (Cronbach’s alpha = .81). The Life Experiences Survey (LES) elicits life events that occurred the last year, e.g., death of a close relative [47]. All events should be rated according to their emotional quality. For the present analysis, the sum of all negatively evaluated events has been included. The retest reliability of this index is sufficient, ranging from r_tt_ = .56 to r_tt_ = .88 [47]. The Anxiety Screening Questionnaire (ASQ) was designed by Wittchen and Boyer [48] for eliciting symptoms related to generalized anxiety disorder and panic disorder. The test score showed high retest reliability and high specificity of detecting anxiety disorders [48]. Lastly, the Prenatal Distress Questionnaire (PDQ) was presented. The questionnaire consists of 12 five-point scales measuring pregnancy-specific distress, e.g., finding the weight gain due to pregnancy troublesome or worrying about eating healthy foods [49]. The total sum score varies from 0 to 48 and showed high correlation (*r* = .53) to global distress [49].

### Procedure

The mothers-to-be were approached during their application visit about 4–8 weeks prior to delivery in three obstetric hospitals in the Rhine-Neckar Region of Germany (Mannheim, Ludwigshafen). They were briefly informed about the study “Pre-, Peri- and Postnatal Stress: Epigenetic impact on Depression; POSEIDON) and received a flyer with a brief outline of the study encompassing three measurement points: third trimester of pregnancy, immediately after delivery and 6 months postpartum. The POSEIDON protocol included interviews, questionnaires, saliva samples, cord blood and placenta tissue sample obtained during delivery, smear tests of the child’s buccal mucosa, diaper urine of the 6 month old child, and a 10 min video session of mother-child interaction. The following inclusion criteria for mothers were applied: Caucasian descent; main caregiver; German-speaking; and age 16–40 years. Exclusion criteria were: maternal hepatitis B, hepatitis C or HIV-infection; any current psychiatric disorders requiring inpatient treatment; a history, current diagnosis of schizophrenia/psychotic disorder, or any substance dependency other than nicotine during pregnancy. Based on rough estimates of deliveries per year within each hospital, it could be estimated that about 33 % of all mothers who met the inclusion/exclusion criteria participated in the study within the recruiting period from October 2010 to March 2013. Participation was reimbursed with 120 Euros. The study protocol was approved by the Ethics Committee of the Medical Faculty Mannheim of the University of Heidelberg, and the study was conducted in accordance with the Declaration of Helsinki. All mothers provided written informed consent prior to participation.

During the third trimester of pregnancy, participants filled in several questionnaires (see questionnaire section) which are included in the present analysis. The present analysis is based on the first measurement point of the larger study.

The representative sample was obtained the following way [33]. The drawing procedure was a three-step process. First, 258 areas were randomly selected (German demoscopic institutes have divided Germany into 53.000 non-overlapping areas with a least 350 households, the ADM sample network). Using again a random procedure every third household was selected. Third, within the household the person (over 14 years.) who was nearest his or her birthday was selected. The study was carried out by Ipsos GmbH, Mölln, Germany. The nightmare questions were part of a multi-themes survey, often about consumer behavior (computer-aided face-to-face interview). The sample size was reduced from 1350 to 915 (496 women, 419 men) because of drop outs due to the following reasons: “not available for the interview” (*N* = 178), refusing to participate (*N* = 172), not completing the nightmare frequency scale (*N* = 72), and other reasons (*N* = 13); i.e., the response rate was 67.8 %.

The statistical analyses were carried out with SAS 9.4 for Windows software. As the nightmare scale and the “dreaming about the baby” item were ordinal, logistic regressions were computed. As age means differed between pregnant women and the representative sample, age was included in the analysis as covariate. For analyzing the intercorrelations between the stress measures a factor analysis (principal component analysis) was carried out.

## Results

The distribution of the eight-point nightmare frequency scale is depicted in Table 1. More than 11 % of the women in the last trimester of their pregnancy reported nightmares once a week or more often. Compared to the representative sample of women (see Table 2), pregnant women in the last trimester reported significantly more nightmares (standardized estimate: .1916, *χ* ^2^ = 21.7, *p* < .0001); controlling for age (standardized estimate: −.1239, *χ* ^2^ = 8.8, *p* = .0030) and dream recall frequency (standardized estimate: .5537, *χ* ^2^ = 189.4, *p* < .0001). Dream recall frequency was chosen as the additional covariate because pregnant women reported more dreams than the representative sample of women (standardized estimate: .2985, *χ* ^2^ = 56.5, *p* < .0001) with age as covariate (standardized estimate: −.1734, *χ* ^2^ = 19.6, *p* < .0001) (see Table 2).

**Table 1:** Nightmare frequency in last trimester of pregnancy (*N* = 397) and in a representative sample of women (*N* = 496)

Category	Pregnant women	Representative sample
Several times a week	4.53 %	0.60 %
About once a week	6.80 %	1.61 %
Twice or three times a month	14.61 %	6.65 %
About once a month	19.40 %	6.45 %
About two to four times a year	21.16 %	10.08 %
About once a year	7.30 %	8.87 %
Less than once a year	7.30 %	19.96 %
Never	18.89 %	45.77 %

**Table 2:** Dream recall frequency and nightmare frequency in pregnant women and a representative sample

Variable	Pregnant women	Representative sample
Dream Recall Frequency	3.64 ± 1.69	2.23 ± 1.77
Dream Recall Frequency (recoded, mornings per week)	2.03 ± 2.15	0.89 ± 1.48
Nightmare Frequency	3.09 ± 2.05	1.41 ± 1.75
Nightmare Frequency (recoded, nightmares per month)	1.71 ± 3.74	0.45 ± 1.59

Means and standard deviations of the stress measures are shown in Table 3. Given the high overlap between the measures, we performed a factor analysis for all seven measures that resulted in one factor (Eigen value > 1 was chosen as criterion) and explained variance of 60.94 %. The factor loadings are depicted in Table 3.

**Table 3:** Stress measures in pregnant women

Variable	Mean ± SD	Factor loadings	Correlation to nightmare frequency
Neuroticism (NEO-FFI)	1.80 ± 0.43	.640	.195***
Perceived Stress Scale (PSS)	21.34 ± 8.45	.848	.267***
Trait anxiety (STAI-T)	36.91 ± 9.80	.872	.298***
Edinburgh Postnatal Depression Scale (EPDS)	6.18 ± 5.47	.881	.296***
Anxiety screening questionnaire (ASQ)	1.78 ± 1.92	.784	.302***
Negative Life experiences (LES)	3.64 ± 3.25	.612	.210***
Prenatal Distress Questionnaire (PDQ)	12.99 ± 7.66	.783	.296***

***p.001

All seven stress measures correlated significantly with nightmare frequency (see Table 3). The logistic regression including age, parity and dream recall frequency indicated that the composed stress factor (factor score of all seven measures) is significantly related to nightmare frequency (see Table 4). Age and parity had no effect.

**Table 4:** Influencing factors of nightmare frequency (logistic regression)

Variable	Standardized estimate	Wald *χ* ^2^	*P* value
Age	−.0130	0.1	.8092
Parity (multiparous vs. nulliparous)	−.0443	0.7	.3961
Composed Stress Factor	.4235	56.9	< .0001
Dream recall frequency	.6036	105.2	< .0001

Most of the pregnant women reported that they dreamed about their baby (see Table 5). Whereas stress was not related to the frequency of baby-related dreams, younger women and nulliparous women dreamed more often about their future babies (see Table 6). Interestingly, higher nightmare frequency was also related to more baby-related dreams; a finding that cannot explained by dream recall frequency because this was included as a possible confounder and, thus, was statistically controlled.

**Table 5:** Item “I dream about the baby” of the Prenatal Attachment Inventory (*N* = 397)

Category	Frequency	Percentage
Almost always	43	10.83 %
Often	68	17.13 %
Sometimes	159	40.05 %
Almost never	127	31.99 %

**Table 6:** Influencing factors of “dreaming about the baby” variable (logistic regression)

Variable	Standardized estimate	Wald *χ* ^2^	*P* value
Age	−.1296	5.4	.0197
Parity (multiparous vs. nulliparous)	−.1078	4.0	.0455
Dream recall frequency	.1070	3.0	.0807
Nightmare frequency	.1551	5.7	.0167
Composed Stress Factor	−.0001	0.0	.9882

## Discussion

The present study clearly indicates that pregnant women report nightmares more often compared to a representative sample and that nightmare frequency is closely related to subjectively experienced stress during daytime. Moreover, baby-related dreams were correlated with nightmare frequency but not with day-time stress.

Even though the present sample of pregnant women was a selected sample (response rate of about 33 % presumably due to the high time expenditure requested by the POSEIDON study protocol), the percentage of women reporting nightmares once a week or more often are much lower (11 % vs. 32 %) than the percentages reported by Lara-Carrasco, Simard [21]. This indicates that the selection criteria regarding dream recall biased the previous findings [21]. Interestingly, pregnant women also recalled their dreams more often than a representative sample but this difference did not affect the difference in nightmare frequency, i.e., pregnant women reported more nightmares regardless of the increased dream recall. The prevalence of the nightmare disorder in the general population is about 5 % [32]. Based on the present percentage of women with frequent nightmares, it would be very interesting to carry out a study including a formal diagnosis regarding the presence of a nightmare disorder [28], i.e., to determine the percentage of pregnant women who suffer from nightmares in clinically significant way.

The medium-sized correlations between nightmare frequency and the single stress measures and the stress index in this sample of pregnant women are comparable with findings in non-pregnant samples [35, 50, 51]. As pregnancy, especially the third trimester, is a period of increased stress [2], it would be very interesting to study whether the increased daytime stress explains the increased number of nightmares during pregnancy. We were unable to do this analysis in the present study as we had no data on daytime stress in the representative sample. Furthermore, it would be very interesting to include measures of nightmare distress, i.e., stress related to the occurrence of nightmares and how this specific stress factor is related to the overall stress of the pregnant woman.

From a methodological viewpoint, it has to be noted that the representative sample used for comparison might have included a small number of pregnant women but, if that had been the case, the results would be even more pronounced if they would have been excluded (as no information was available, it could not be done in the present analysis). Another topic discussed in the literature is the higher number of nightmares obtained using diary measures compared to retrospective questionnaires [52–54]. I.e., the present prevalence rate might be higher if daily measures had been used. As the effect size of the difference between the retrospective and prospective measures is relatively small (*d* = 0.101; [55]), one would not expect much higher rates (e.g., as reported in [21]) but nevertheless, it would be very interesting to carry out diary studies to elicit nightmare frequency in pregnant women, especially in regard to determining the percentage of women with nightmare disorders. As nightmares might be confused with night terrors, nocturnal panic attacks and so on it would be advisable to include a specific definition for nightmares (second part of the night, good recall); even though the possible bias is very small as NREM parasomnias and nocturnal panic attacks are very rare compared to nightmares [32]. Lastly, the present analysis is part of a larger study which required a lot of time and effort from the participants and, thus, resulted in a relatively low response rate. The advantage was that there was no selection regarding dreaming or nightmares that might have occurred if pregnant women were approached with information sheets about a dream/nightmare study. But, one can imagine that highly stressed women were less likely to participate in an extensive study and, thus, it would make sense to devise a simpler protocol, maybe aiming at sleep, in order to obtain a higher response rate.

As reported previously (e.g., [8]) that pregnant women dream about their baby-to-be, about two-thirds in our sample reported baby-related dreams. Nulliparous women reported those dreams more often than multiparous women – a finding which is in line with the results of Blake and Reimann [13]. In addition, younger women also reported more often baby-related dreams, i.e., one might expect that younger women are more stressed during pregnancy [56]. The significant correlation between nightmare frequency and the frequency of baby-related dreams indicate that those negatively-toned dreams reflect day-time worries about the health of the fetus which would be in line with previous findings [8].

## Conclusions

To summarize, the findings clearly indicated a heightened nightmare frequency in pregnant women in their last trimester. Thus, questions about nightmare complaints should be included into the routine medical care of pregnant women. As insomnia during pregnancy might be a predictor for post-partum depression [57], it might be interesting to study whether frequent nightmares during pregnancy might have some predictive value with respect to postpartum mental disorders, including posttraumatic stress disorder due to childbirth [58]. One study [59] found that women developing a postpartum depression reported fewer negative dreams (56 % vs. 32.3 %) than women who did not. As dream recall frequency is often reduced in depression [60], this finding might be different if dream recall frequency had been controlled. If a substantial number of pregnant women suffer from nightmare disorders it would be very interesting to learn whether short-term interventions like Imagery Rehearsal Therapy [61] that have been shown to be very effective in different samples [62] are also beneficial for pregnant women with nightmares. Imagery rehearsal therapy is based on principles of cognitive therapy and encompasses three steps: Confrontation (recording the dream), Coping (imagining a new, more satisfying dream ending) and Rehearsal (imagining the new coping strategy once a day for 5–10 min over 2 weeks). Lastly, it would be very interesting to carry out longitudinal studies since the only study [29] reporting prepregnant values of nightmare frequencies elicited retrospectively reported a slight decrease in nightmare frequency due to pregnancy.

## Abbreviations

ASQ: Anxiety screening questionnaire
EPDS: Edinburgh postnatal depression scale
LES: Life experiences survey
NEO: Neuroticism extraversion openness to experience
PDQ: Prenatal distress questionnaire
POSEIDON: Pre-, peri- and postnatal stress: epigenetic impact on depression
PSS: Perceived stress scale
SAS: Statistical analysis system
STAI: State-trait-anxiety inventory
